# An Electrochemical Sensor of Theophylline on a Boron-Doped Diamond Electrode Modified with Nickel Nanoparticles

**DOI:** 10.3390/s23208597

**Published:** 2023-10-20

**Authors:** Prastika Krisma Jiwanti, Anis Puspita Sari, Siti Wafiroh, Yeni Wahyuni Hartati, Jarnuzi Gunlazuardi, Yulia M. T. A. Putri, Takeshi Kondo, Qonita Kurnia Anjani

**Affiliations:** 1 Nanotechnology Engineering, Faculty of Advanced Technology and Multidiscipline, Universitas Airlangga, Surabaya 60115, Indonesia; 2 Department of Chemistry, Faculty of Science and Technology, Universitas Airlangga, Surabaya 60115, Indonesiasitiwafiroh@fst.unair.ac.id (S.W.); 3 Department of Chemistry, Faculty of Mathematics and Natural Sciences, Universitas Padjadjaran, Jatinangor 45363, Indonesia; yeni.w.hartati@unpad.ac.id; 4 Department of Chemistry, Faculty of Mathematics and Natural Sciences, Universitas Indonesia, Kampus UI Depok, Jakarta 16424, Indonesia; jarnuzi.gunlazuardi@sci.ui.ac.id (J.G.); yulia.mariana@sci.ui.ac.id (Y.M.T.A.P.); 5 Department of Pure and Applied Chemistry, Tokyo University of Science, 2641 Yamazaki, Noda 278-8510, Chiba, Japan; t-kondo@rs.tus.ac.jp; 6 School of Pharmacy, Medical Biology Centre, Queen’s University Belfast, 97 Lisburn Road, Belfast BT9 7BL, UK; qonita.anjani@qub.ac.uk

**Keywords:** theophylline, boron-doped diamond, nickel nanoparticles, electrochemical sensing, human health

## Abstract

Theophylline is a drug with a narrow therapeutic range. Electrochemical sensors are a potentially effective method for detecting theophylline concentration to prevent toxicity. In this work, a simple modification of a boron-doped diamond electrode using nickel nanoparticles was successfully performed for a theophylline electrochemical sensor. The modified electrode was characterized using a scanning electron microscope and X-ray photoelectron spectroscopy. Square wave voltammetry and cyclic voltammetry methods were used to study the electrochemical behavior of theophylline. The modified nickel nanoparticles on the boron-doped diamond electrode exhibited an electrochemically active surface area of 0.0081 cm^2^, which is larger than the unmodified boron-doped diamond’s area of 0.0011 cm^2^. This modified electrode demonstrated a low limit of detection of 2.79 µM within the linear concentration range from 30 to 100 µM. Moreover, the modified boron-doped diamond electrode also showed selective properties against D-glucose, ammonium sulfate, and urea. In the real sample analysis using artificial urine, the boron-doped diamond electrode with nickel nanoparticle modifications achieved a %recovery of 105.10%, with a good precision of less than 5%. The results of this work indicate that the developed method using nickel nanoparticles on a boron-doped diamond electrode is promising for the determination of theophylline.

## 1. Introduction

Theophylline is an alkaloid compound that is effective for treating asthma and has been used for over 80 years [1]. Theophylline is consumed as a bronchodilator agent for asthma and obstructive chronic pulmonary disorders [2]. Theophylline works through adenosine receptors like A1, A2, and A3 by acting as a non-selective antagonist [3]. Theophylline is a drug that has a narrow therapeutic window. Therefore, the concentration of theophylline really needs to be monitored to prevent the drug’s toxicity. In the plasma of adults, the safety concentration of theophylline ranges from 10 to 20 µg/mL, and for children, it ranges from 5 to 10 µg/mL [4]. A concentration of theophylline that is more than 20 µg/mL can cause toxicity symptoms, such as severe nausea and vomiting, cardiac arrythmias, hypotension, and seizures [5].

There are several previous methods that have been used to determine theophylline, including high performance liquid chromatography (HPLC) [6], fluorescence [7], and spectroscopy [8]. However, these methods necessitate individual abilities, sample preparation, and lengthy analysis. In 2018, Chen et al. reported that RNA aptamers modified with gold nanoparticles (AuNP) can be used as theophylline sensors [9]. Nevertheless, this method was complicated, and to determine theophylline, it is necessary to use a simple method with a good response. The electrochemical method is a good choice for determining theophylline due to its advantages, which are more sensitivity, good stability, ease of operation, and quick response [10]. The most commonly used working electrode in the electrochemical method is carbon, which has high conductivity, stability, and a low background current compared to other materials [11].

Many working electrodes have been used as electrochemical sensors, such as glassy carbon (GC), pencil graphite carbon, and screen-printed electrodes. Boron-doped diamond (BDD) can also be used as a working electrode because of its advantages, which include a high potential window, high density, low background current, and good stability both physically and chemically. Besides being applied as a sensor, BDD is also widely applied for fuel cell construction, CO_2_ reduction, and electrosynthesis [12]. As a theophylline electrochemical sensor, unmodified BDD has demonstrated an excellent %recovery in real samples, including urine and pharmaceutical drugs, of 93.2–102.5% [13]. On the other hand, many researchers have described various modifications to the BDD surface for enhancing the electroanalytical performance when sensing using BDD [12].

Metal nanoparticles have become widely studied by many researchers because of their characteristics as electrocatalysts. BDD modified with metal material provides some advantages, such as a low LOD and a large surface area [14,15,16]. Noble metal nanoparticles, including gold nanoparticles (AuNPs), platinum nanoparticles (PtNPs), and palladium nanoparticles (PdNPs), have been used for sensors, and have excellent electrocatalytic activities and good antifouling abilities. However, these materials have limited availability, are not sustainable, and are toxic [17]. Thus, non-precious metal nanoparticles are needed for use as modification materials and can be used sustainably. Nickel is one of the alternative metals that can be applied as a modification material due to its low price, abundance in nature, low toxicity, and stability, which is similar to that of noble metal particles [18,19]. In a previous report, nickel nanoparticles (NiNPs) were used as a modification material and successfully performed as a high-sensitivity and low-detection-limit (LOD) sensor for levofloxacin, glucose, and bisphenol A [18,19,20,21]. Subsequently, NiNPs offer excellent properties as an electrocatalyst, which can enhance sensor performance. Thus far, there have been no reports on the modification of BDD with NiNPs for theophylline sensing. In this work, NiNPs and BDD electrodes will be developed as a simple modification method of determining theophylline compounds with a low LOD, selective properties, and good %recovery.

## 2. Materials and Methods

### 2.1. Chemicals and Instruments

NaH_2_PO_4_ (>99%) and Na_2_HPO_4_ (>99.5%) were purchased from Millipore Corporation, Burlington, MA, USA. NiSO_4_ (>99%), CH_3_COOH (≥99.7%), CH_3_COONa (≥99%), and theophylline (>99%) were purchased from Sigma Aldrich (Dorset, UK). H_2_SO_4_ (>98%) and ultrapure water were purchased from SAP Chemical, Jakarta, Indonesia. All chemicals were used without further purification.

The Emstat^3+^ Blue Palmsens potentiostat was used to measure voltammetric data with an Ag/AgCl electrode as a reference electrode, and platinum wire was used as an auxiliary electrode. X-ray photoelectron spectroscopy (XPS) (JPS-9010TR, JEOL, Tokyo, Japan) and scanning electron microscopy (SEM) (JCM-6000, JEOL, Japan) were used to characterize the modified electrode.

### 2.2. Preparation of the Modified BDD Electrode

Pretreatment of the BDD electrode was needed before modification with nickel nanoparticles. The BDD electrode was sonicated in 1-propanol and ultrapure water for 5 min each. After that, the BDD electrode was optimized in 0.1 M H_2_SO_4_ via CV with a scan rate of 1 V/s and 40 scans in the potential range of −2.0 V to +2.0 V.

Modification of the BDD electrode using NiNPs was carried out using the electrodeposition method. NiSO_4_ 1 mM, used as a precursor of Ni^2+^, was deposited onto the BDD surface using chronoamperometry, with a potential of −1.2 V. The solution was added to a 5 mL electrochemistry cell that contained 0.1 M acetic buffer solution at pH 5.5, and deposition ran for 250 s. This modified electrode was then characterized using SEM and XPS.

### 2.3. Measurement Procedure

The concentration of theophylline was evaluated by adding 60 µM of theophylline stock solution (1 mM) to an electrochemical cell with 0.1 M PBS as a supporting electrolyte. CV and SWV methods were performed to determine theophylline using an Emstat^3+^ Blue Palmsens potentiostat. The LOD of theophylline was calculated as three times the standard deviation of the blank divided by the slope. On the other hand, the real sample was analyzed using the standard addition method.

### 2.4. Sample Preparation for Real Sample Analysis

The analysis of the real sample was performed on artificial urine. The solution of artificial urine was prepared by dissolving 0.73 g NaCl, 0.40 g KCl, 0.27 g CaCl_2_·2H_2_O, 0.56 g Na_2_SO_4_, 0.35 g KH_2_PO_4_, 0.25 g NH_4_Cl, and 6.25 g urea in 250 mL of distilled water [22]. An aliquot of artificial urine was added to the electrochemical cell and then spiked with 0.3 mL of theophylline stock solution (1 mM). The solution was then measured in 0.1 M phosphate buffer using the SWV method under optimum conditions.

## 3. Results and Discussion

### 3.1. Characterization of the BDD/NiNP Electrode

**Figure 1:**
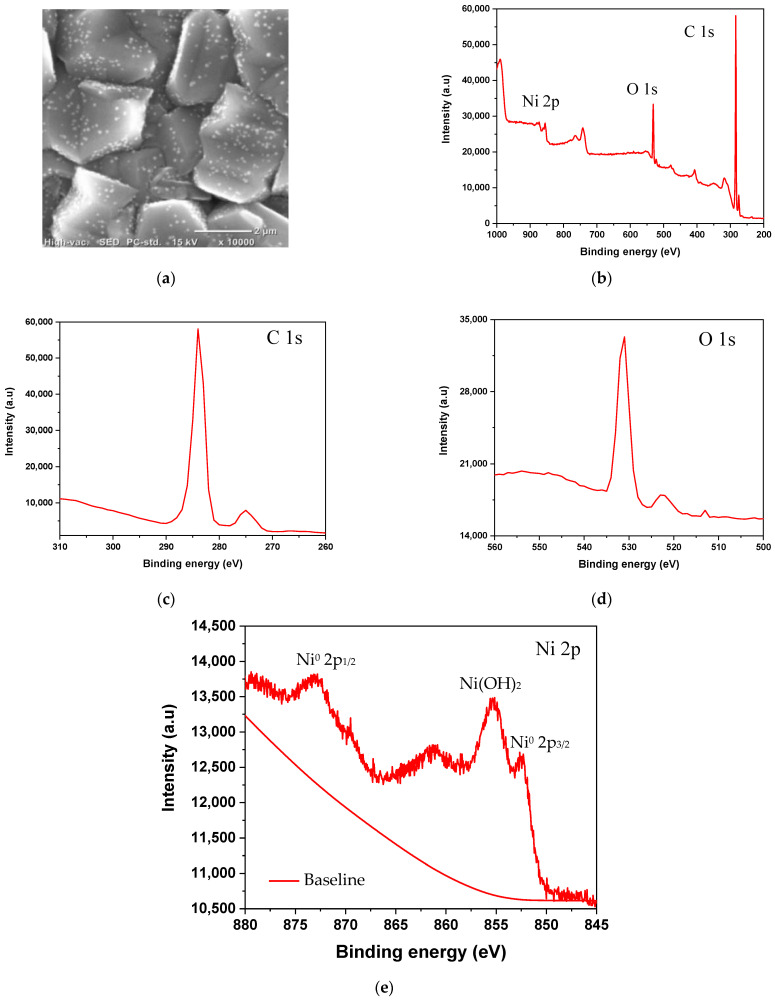
(**a**) SEM images of BDD/NiNP, (**b**) XPS spectra of BDD/NiNP (wide scan), (**c**) C 1s spectra, (**d**) O 1s spectra, and (**e**) Ni 2p spectra with baseline (red line).

The surface topography of the BDD/NiNP electrode is presented using SEM instruments. Figure 1a shows the SEM image of the electrode and nickel deposition quite homogeneously on the BDD surface. The average size of the deposited nickel particles is 82.27 nm, which was processed using ImageJ version 1.53t and OriginPro software 2018. Moreover, the BDD/NiNP electrode was characterized using XPS to study the elemental composition of the BDD/NiNP, as shown in Figure 1b. 

The XPS narrow spectra in Figure 1c–e show that there is a Ni 2p element that has been identified, indicating that nickel has been successfully deposited on the BDD surface. The spectral peaks at 852.8 eV and 873 eV represent the binding energies of Ni species (Ni^0^) in the 2p_3/2_ and 2p_1/2_ orbitals [18]. Another spectral peak appears at a binding energy of 855.37 eV that represents Ni(OH)_2_ [23]. These XPS data show that the surface of the BDD/NiNP electrode contains Ni^0^ (metallic Ni), along with Ni^2+^ species, which form Ni(OH)_2_. The deconvoluted C 1s spectrum shows a significant peak at a binding energy of around 283 eV [18]. Furthermore, the peak of the spectra at 531.0 eV is a spectrum of O 1s, which represents the presence of a bond between a metal and hydroxide (M-OH) [24]. In addition, according to the XPS data, the ratio of each identified element is 4.08% Ni 2p_3/2_, 8.18% Ni 2p_1/2_, 86.71% element C, and 1.03% element O.

### 3.2. Electrochemical Study of BDD and BDD/NiNP

The electrochemically active surface area of BDD/NiNP was studied using a [Fe(CN)_6_]^3−^/[Fe(CN)_6_]^4−^ solution. The Randles–Sevcik equation was used to calculate it [18]:

*I_pa_* = (2.69 × 10^5^) × *n*^3/2^ × *D*^1/2^ × *v*^1/2^ × *A*× *C* (1)

where *I_pa_* is the peak current (A), *n* is the number of electrons transferred, *D* is the diffusion coefficient of K_3_Fe(CN)_6_ (7.6 × 10^−6^ cm^2^/s), *v* is the scanning rate, *A* is the electrochemically active surface area (cm^2^), and *C* is the concentration of bulk solution (5 mM). An aliquot of K_3_Fe(CN)_6_ was used with 0.1 M KCl as an electrolyte solution. The CV method was applied at various scan rates from 40 to 120 mV/s.

**Figure 2:**
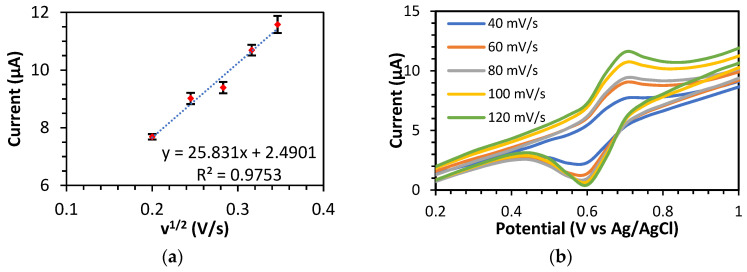
(**a**) A plot of the square root of the scan rate vs. peak oxidation current (red dot) with error bar (black line) and (**b**) CV curves for various scan rates from 40 to 120 mV/s in an aqueous solution of 5.0 mM K_3_[Fe(CN)_6_] with 0.1 M KCl using BDD/NiNP.

The redox peak current of the K_3_Fe(CN)_6_ solution in the 0.1 M KCl electrolyte on the electrode increases as the scan rates increase. The slope of the plotting curve of the square root of the scan rate against the peak current for BDD/NiNP (Figure 2) was used to calculate the electrochemically active surface area. Following the equation, the electrochemically active surface area of BDD/NiNP was estimated at 0.0081 cm^2^, which is eight times larger than the surface area of unmodified BDD (0.0011 cm^2^) (Figure S1). The modification of the nanoparticles on the surface of the BDD electrode results in a larger active surface area and, thus, better electron transfer, indicating that the sensor has enhanced electroanalytic capabilities in detecting theophylline.

**Figure 3:**
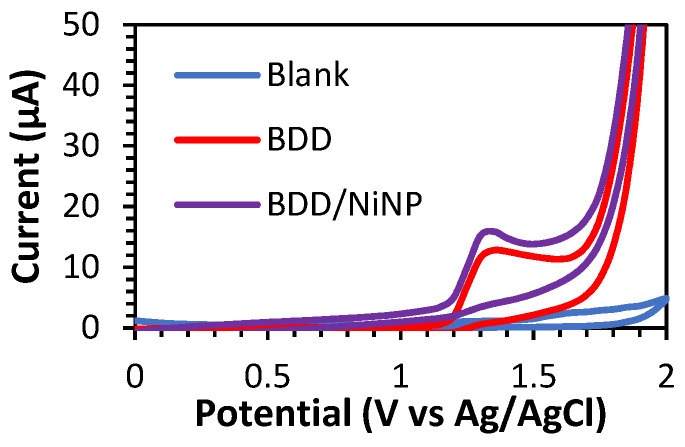
CV curves of the blank (0.1 M PBS) for the BDD electrode (blue), and CV curves for BDD (red) and BDD/NiNP electrodes (purple) in 60 µM theophylline at a scan rate of 100 mV/s.

The BDD and BDD/NiNP electrodes were also characterized via cyclic voltammetry (CV) for theophylline determination. As shown in Figure 3, the peak of theophylline oxidation appears within a potential range of +1.20 V (vs. Ag/AgCl) to +1.50 V (vs. Ag/AgCl), and there are no peak currents in the reverse scan, indicating that theophylline oxidation is irreversible. The CV curves of BDD and BDD/NiNP were determined using 60 µM theophylline with a scan rate of 100 mV/s. According to reports, the oxidation peak of theophylline on BDD/NiNP occurred at 15.89 µM and 12.80 µM for BDD, respectively. The higher peak current on BDD/NiNP compared to unmodified BDD indicates that the NiNP material enhances the oxidation of theophylline as the electrocatalytic activity increases.

### 3.3. Electrochemical Performance of Theophylline

#### 3.3.1. Determination of Signal Per Background (S/B)

**Figure 4:**
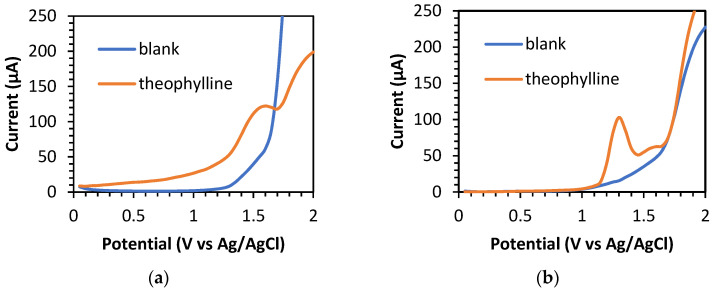
SWV curves for determining S/B of 60 μM theophylline in 0.1 M PBS pH 3, (SWV parameters: frequency of 50 Hz, step potential of 50 mV, and amplitude of 50 mV) using (**a**) BDD and (**b**) BDD/NiNP electrodes.

The S/B measurement provides information about the ratio between the background current and the sample analysis current. A larger ratio indicates that the detected background current is very small and can be ignored, thus affecting the low detection limit of the electrode [14]. The determination of S/B was recorded over a potential range of 0 to 2.0 V (vs. Ag/AgCl), with the optimum parameters of SWV (Figure S2), by comparing the BDD and BDD/NiNP electrodes. PBS at pH 3.0 was used as the blank for this determination. Theophylline was successfully detected on both electrodes at a potential of +1.60 V (vs. Ag/AgCl) on the BDD and +1.30 V (vs. Ag/AgCl) on the BDD/NiNP electrodes, respectively (Figure 4). The BDD/NiNP electrodes produced a greater S/B value of 6.63 than the BDD electrode’s value of 1.98. The NiNP modification on the surface of the electrode causes the background current to be much smaller (15.53 µA) than on the unmodified electrode (61.64 µA), and results in better quality detection of low concentrations of theophylline.

#### 3.3.2. Effect of Scan Rate

**Figure 5:**
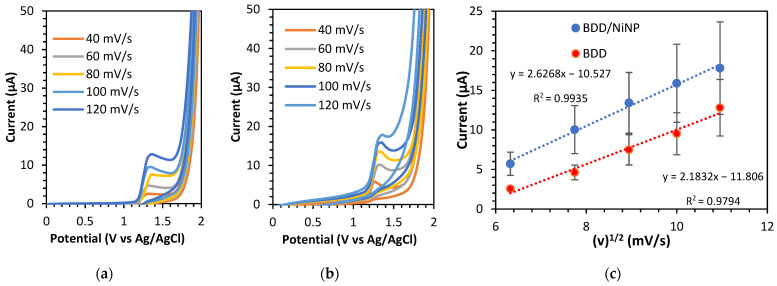
CV curve for various scan rates from 40 to 120 mV/s, using (**a**) BDD and (**b**) BDD/NiNP electrodes. (**c**) Linear plot of the oxidation peak current vs. square root of the scan rates in measuring 60 μM theophylline with 0.1 M PBS at pH 3 at various scan rates from 40 to 120 mV/s using BDD (red dot) and BDD/NiNP (blue dot).

Additionally, CV was used to examine theophylline’s electrochemical oxidation in 0.1 M PBS at various scan rates, ranging from 40 mV/s to 120 mV/s. The oxidation peak currents for the BDD and BDD/NiNP electrodes increase as the scan rates increase. During the reverse current, there is no visible peak current, and the potential shifts to a more positive potential, indicating that theophylline is categorized as a compound that undergoes an irreversible oxidation reaction [25]. Moreover, the results of the linear plot between the oxidation peak current and the square root of the scan rates, as shown in Figure 5, provide evidence that theophylline oxidation activity on the electrode surface undergoes a diffusion-controlled process [26].

#### 3.3.3. Effect of pH

**Figure 6:**
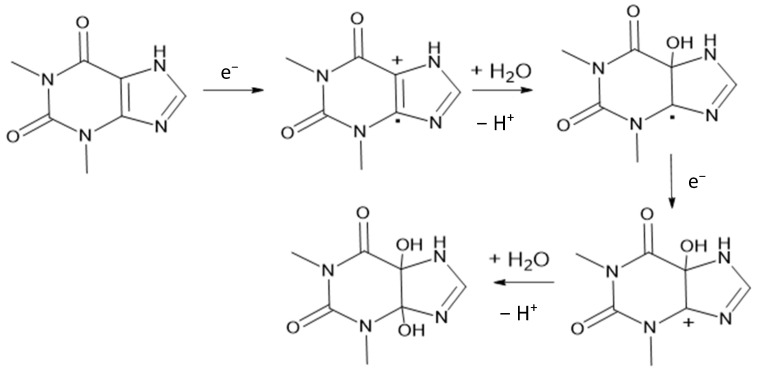
The proposed mechanism of theophylline oxidation [28].

**Figure 7:**
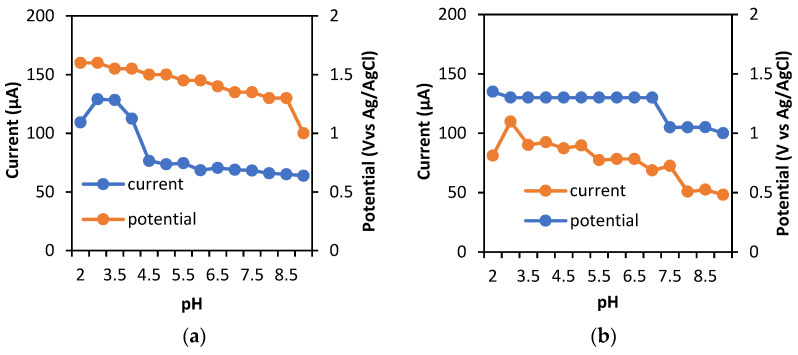
Graph of the relationship between pH, potential, and current in 60 μM theophylline measurements with 0.1 M PBS pH 2–9 using (**a**) BDD and (**b**) BDD/NiNP, (SWV parameters: frequency of 50 Hz, step potential of 50 mV, and amplitude of 50 mV).

The pH value is determined to investigate the effect of pH on the current and the potential for theophylline oxidation. Various pHs of 0.1 M PBS, from 2 to 9, were applied to the BDD and BDD/NiNP electrodes. The potential shift towards a more negative potential increased with the pH. The proposed mechanism (Figure 6) involves the transfer of two protons and two electrons during the theophylline oxidation reaction, which affects the optimum pH value obtained. In low-pH conditions, the protonation of theophylline oxidation is favorable, resulting in a high potential, which is required for the oxidation of theophylline [27]. Furthermore, the highest current response in each electrode was found at pH values of 3.0, and decreased at higher pH values (Figure S3). Thus, it was selected as the optimum pH for this work. The relationship between the pH, potential, and current of theophylline on BDD and BDD/NiNP electrodes is shown in Figure 7.

#### 3.3.4. Linearity and Detection Limits of Theophylline

**Figure 8:**
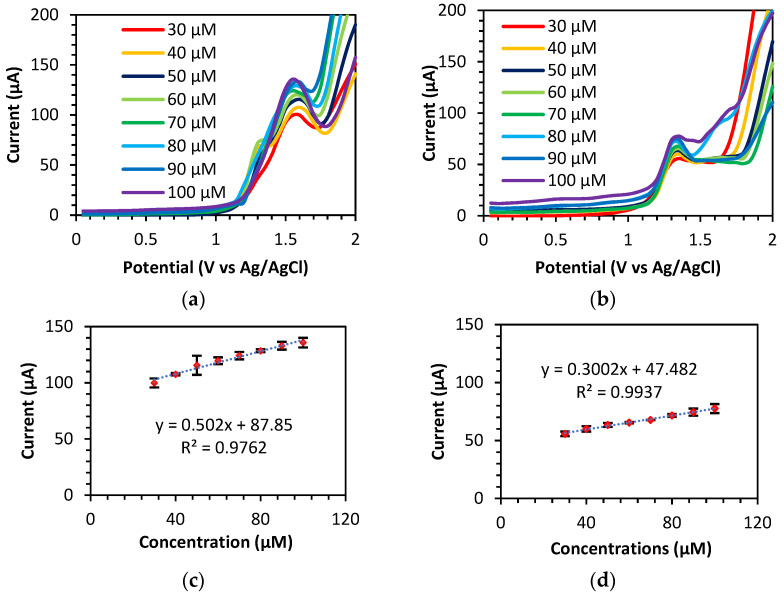
SWV curve using (**a**) BDD and (**b**) BDD/NiNP in various concentrations of theophylline from 30 to 100 μM with 0.1 M PBS at pH 3 (SWV parameters: frequency of 50 Hz, step potential of 50 mV, and amplitude of 50 mV). Plot calibration curves between peak current and concentrations in red dot with error bar (black line) using (**c**) BDD and (**d**) BDD/NiNP.

The SWV method was used to study the effect of various concentrations of theophylline on the peak current response. A range of theophylline concentrations from 30 to 100 µM were used in this work. Subsequently, the peak current of theophylline on the BDD and BDD/NiNP electrodes increases as the concentrations of theophylline increase. The slope of the calibration curve of the various theophylline concentrations against the peak current (Figure 8c,d) was used to calculate the limit of detection (LOD) of theophylline.

The LOD of theophylline was estimated by using the following equation:

*LOD* = 3 × *Sa*/*m* (2)

where *Sa* is the blank standard deviation, and *m* is the slope of the calibration curve [29]. The BDD and BDD/NiNP electrodes demonstrate detection limits of 4.58 μM and 2.79 μM within the linear concentration range of 30–100 μM, respectively. The BDD/NiNP electrode exhibits a lower LOD than the unmodified BDD electrode, proving the advantageous properties of metal nanoparticles. The obtained results are compared to the results of previous work, as shown in Table 1. Hence, the BDD and BDD/NiNP electrodes exhibit higher sensitivity than the electrodes in previous work. Although the sensitivity of the BDD/NiNP electrode is less than the sensitivity of the unmodified BDD electrode, BDD/NiNP has a lower background current, which contributes to the low LOD. Subsequently, BDD/NiNP electrodes are expected to detect theophylline at lower concentrations than BDD electrodes. In comparison with previous works, the modification of BDD electrodes with NiNPs offers a simple modification with good sensitivity, and is a promising method for determining that theophylline is within a safe concentration range in the plasma of patients (55 to 110 μM) [30].

#### 3.3.5. Selectivity and Reproducibility

Urea, D-glucose, and ammonium sulfate were used to study interference in the determination of theophylline. These compounds could potentially interfere with the determination of theophylline in the real sample. The selectivity was investigated by combining the theophylline mixture with the interference solution at a concentration ratio of 1:1. Consequently, none of the interference compounds produced a peak current response (Figure S4). The presence of the interfering compounds has no effect on the oxidation peak of theophylline. Thus, both the BDD and BDD/NiNP electrodes are indicated to be selective for urea, D-glucose, and ammonium sulfate in the determination of theophylline.

Reproducibility is important in determining the precision of a method. When using the SWV method, the peak current response of each electrode does not change significantly (Figure S5). The %RSD (*n* = 8) on the BDD electrode is estimated to be 2.69% and approximately 1.36% on the BDD/NiNP electrode, respectively. The %RSDs are less than 5%, which indicates that the two electrodes have a fairly good level of precision and stability. A summary of the validation parameters is presented in Table 2.

### 3.4. Real Sample Analysis

**Figure 9:**
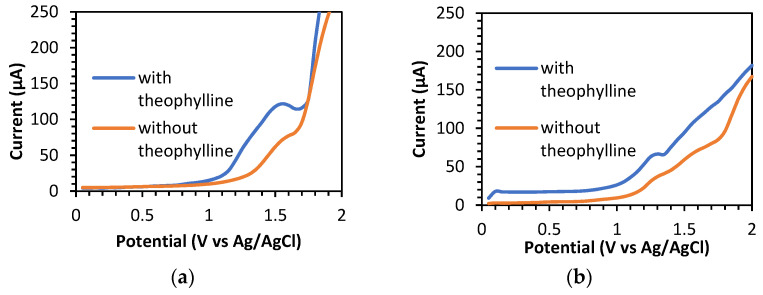
SWV curve using (**a**) BDD and (**b**) BDD/NiNP in an artificial urine sample spiked with 60 μM theophylline, with 0.1 M PBS at pH 3 (SWV parameters: frequency of 50 Hz, step potential of 50 mV, and amplitude of 50 mV).

The application of the developed method using BDD/NiNP and unmodified BDD electrodes was studied using artificial urine. The preparation of the solution is mentioned in Section 2.4, and recovery was examined using the addition standard method. No peak current appeared in the artificial urine analysis. Hence, an aliquot of theophylline stock solution was added to the solution (Figure 9), and the analysis of the sample was carried out three times. Table 3 shows that the recovery of theophylline in both electrodes ranges from 99.87 to 105.10%, with the RSD values being lower than 5%. These results demonstrate that the developed electrochemical method has good accuracy and is promising for the determination of theophylline in future real sample applications.

## 4. Conclusions

The XPS and SEM characterizations showed that the BDD/NiNP electrode was successfully prepared as a theophylline electrochemical sensor using the electrodeposition method. The NiNP modification of the BDD surface resulted in an electrochemically active surface area of 0.0081 cm^2^, which is eight times larger than that of unmodified BDD (0.0011 cm^2^), thus contributing to the increased electroanalytical performance of the sensor. The S/B of theophylline using BDD/NiNP also demonstrated a lower background current than unmodified BDD. The scan rate analysis of both electrodes shows that the oxidation of theophylline is an irreversible process. The optimum pH of theophylline (pH 3) involves two protons and two electrons in the oxidation reaction. The BDD/NiNP electron exhibited a lower LOD, found at 2.79 µM, than the unmodified BDD electron, found at 4.58 µM, within a linear concentration range from 30 to 100 µM. Moreover, both electrodes were selective for D-glucose, ammonium sulfate, and urea, respectively. An acceptable recovery, ranging from 99.87 to 105.10%, and good precision of less than 5% shows that BDD and BDD/NiNP electrochemical sensors are acceptable methods for the detection of theophylline in real sample applications. 

## Figures and Tables

**Table 1:** Comparison of theophylline electrochemical sensors with other previous works.

Electrodes	Concentration Range (μM)	LOD (μM)	Sensitivity (μA/μM)	References
NiO/MWCNT/NNaM/PGE	5–200	0.361	0.457	[21]
3D NiO-NWS/GCE	0.1–900	0.03	0.0049	[29]
SPE/GQD	1–700	0.2	0.0299	[10]
GC/Poly(PABSA)	10–100	7.02	0.224	[31]
GC/Cysteic acid	2.5–68	1.2	0.0036	[32]
BBFT/IL/GPE	12–1200	9.2	0.01	[33]
SPE	55–290	10	0.012	[30]
MWNTs/Au/PLL SPE	10–200	2.0	0.0095	[34]
BDD/NiNP	30–100	2.79	0.3002	This work
BDD	30–100	4.58	0.5020	This work

Acronyms: NiO/MWCNT/NnaM/PGE = nickel oxide/multi-walled carbon nanotube/nano-Na-montmorillonite clay/pencil graphite electrode, 3D NiO-NWS/GCE = three-dimensional nickel oxide nanowrinkles/glassy carbon electrode, SPE/GQD = screen-printed electrode/graphene quantum dot, GC/Poly(PABSA) = glassy carbon/poly(PABSA), BBFT/IL/GPE = 1–4(bromobenzyl)-4-ferrocenyl-1H-[1,2,3]-triazole (1,4-BBFT)/ionic liquids/graphene paste electrode, MWNTs/Au/PLL/SPE = multi-walled carbon nanotubes/Au/poly-L-lysine/SPE.

**Table 2:** A summary of the validation parameters for the determination of theophylline using BDD and BDD/NiNP electrodes.

Parameter Validation	Electrodes	
	BDD	BDD/NiNP
S/B	1.98	6.63
pH Optimum	3.0	3.0
Electrochemical surface-active area (cm2)	0.0011	0.0081
Sensitivity	0.5020	0.3002
LOD (µM)	4.58	2.79
%RSD	2.69	1.36

**Table 3:** Theophylline detection in artificial urine samples.

Electrodes	Added (μM)	Found (μM)	%Recovery	%RSD
BDD	60	59.92	99.87%	4.10%
BDD/NiNP	60	63.05	105.10%	1.65%

## Supplementary Materials

The following supporting information can be downloaded at: https://www.mdpi.com/article/10.3390/s23208597/s1, Figure S1: CV curve of 5.0 mM K_3_Fe(CN)_6_ solution in 0.1 M KCl electrolyte on the BDD electrode; Figure S2: determination of optimum parameters of SWV; Figure S3: SWV curve of various pH (2–9) on BDD and BDD/NiNP; Figure S4: SWV curve of selectivity theophylline with D-glucose, ammonium sulfate, and urea on BDD and BDD/NiNP; Figure S5: SWV curve of reproducibility theophylline on BDD and BDD/NiNP.
