# Supplementary material for: An Electrochemical Sensor of Theophylline on a Boron-Doped Diamond Electrode Modified with Nickel Nanoparticles
Source: Sensors (Basel). 2023 Oct 20;23(20):8597. doi: 10.3390/s23208597 (PMC10611131; doi:10.3390/s23208597)
Supplement: Supplementary file 1 [file sensors-23-08597-s001.zip › sensors-2595091-supplementary.pdf]

Supplementary Data

An Electrochemical Sensor of Theophylline on a Boron-Doped Diamond Electrode Modified with Nickel Nanoparticles

Prastika Krisma Jiwanti [1,*], Anis Puspita Sari [2], Siti Wafiroh [2], Yeni Wahyuni Hartati [3], Jarnuzi Gunlazuardi [4], Yulia M T A Putri [4], Takeshi Kondo [5] and Qonita Kurnia Anjani [6]

[1]Nanotechnology Engineering, Faculty of Advanced Technology and Multidiscipline, University of Air-langga, Surabaya 60115, Indonesia

[2]Department of Chemistry, Faculty of Science and Technology, Universitas Airlangga, Surabaya 60115, Indonesia; sitiwafiroh@fst.unair.ac.id (S.W.)

[3]Department of Chemistry, Faculty of Mathematics and Natural Sciences, Universitas Padjadjaran, Jatinangor 45363, Indonesia; yeni.w.hartati@unpad.ac.id

[4]Department of Chemistry, Faculty of Mathematics and Natural Sciences, Universitas Indonesia, Kampus UI Depok, Jakarta 16424, Indonesia; jarnuzi.gunlazuardi@sci.ui.ac.id (J.G.); yulia.mariana@sci.ui.ac.id (Y.M.T.A.P.)

[5]Department of Pure and Applied Chemistry, Tokyo University of Science, 2641 Yamazaki, Chiba, Noda 278-8510, Japan; t-kondo@rs.tus.ac.jp

[6]School of Pharmacy, Queen's University Belfast, Medical Biology Centre, 97 Lisburn Road, Belfast BT9 7BL, UK; qonita.anjani@qub.ac.uk

*Correspondence: prastika.krisma@ftmm.unair.ac.id

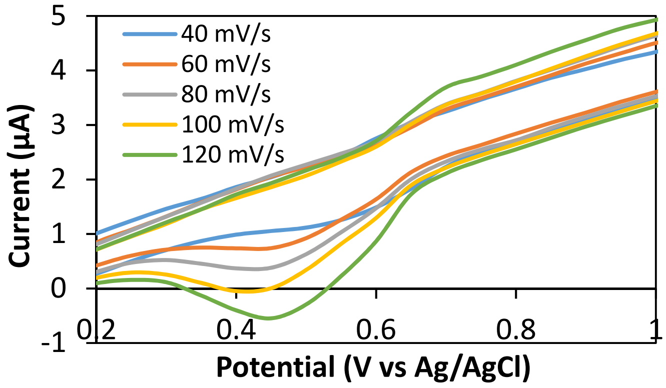

(a)

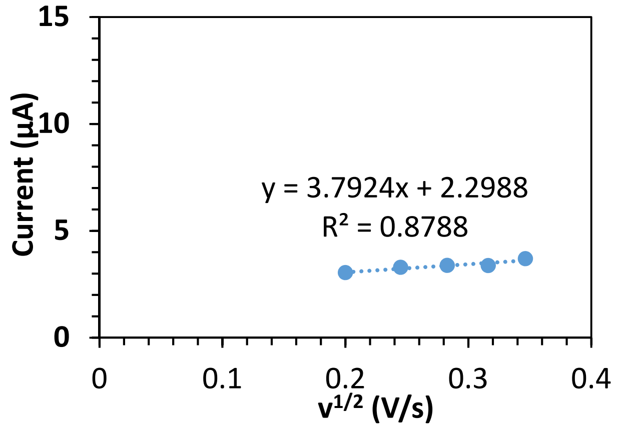

(b)

Figure S1. **(a)** CV curve of variation scan rates ranges from 40 to 120 mV/s for 5.0 mM [$K_3Fe(CN)_6$] in 0.1 M KCl electrolyte and (b) Plot of the square root of scan rate vs peak current using BDD electrode.

scan rate (V/s)	Square root of the *scan rate* (V/s)	Peak current (A)	BDD/NiNP peak current (A)
0.04	0.2	0.00000304	0.000007686
0.06	0.244948974	0.000003286	0.000009018
0.08	0.282842712	0.000003379	0.000009392
0.1	0.316227766	0.00000337	0.00001069
0.12	0.346410162	0.000003692	0.00001158

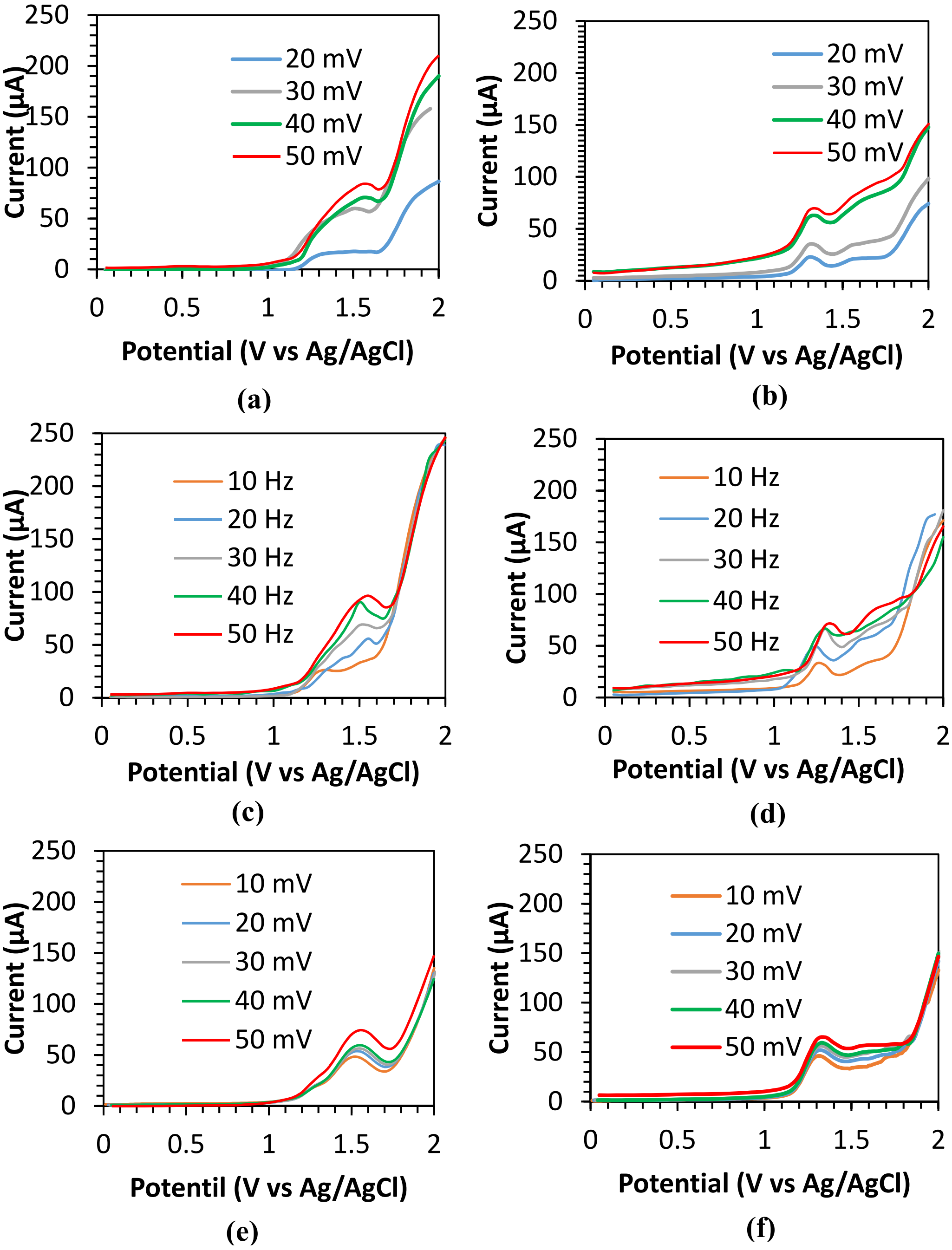

Figure S2. SWV curve of optimum amplitude determination using **(a)** BDD and **(b)** BDD/NiNP, optimum frequency determination using **(c)** BDD and **(d)** BDD/NiNP, optimum step potential determination using **(e)** BDD and **(f)** BDD/NiNP in measuring 60 µM theophylline with 0.1 M PBS pH 3.

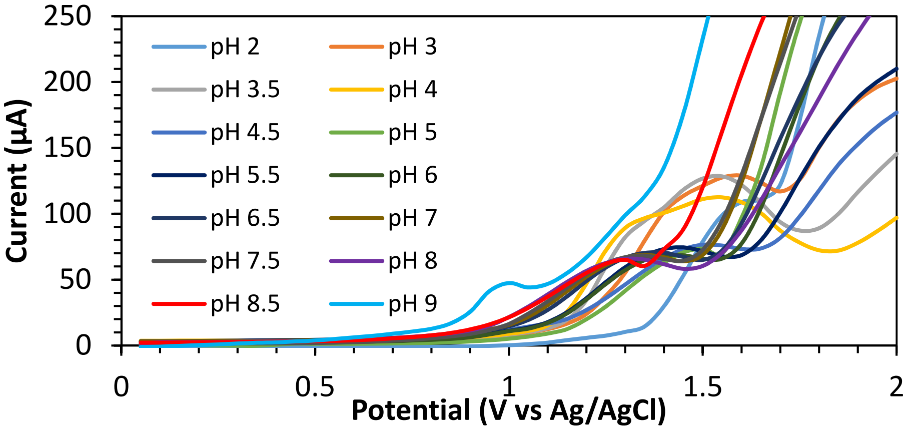

(a)

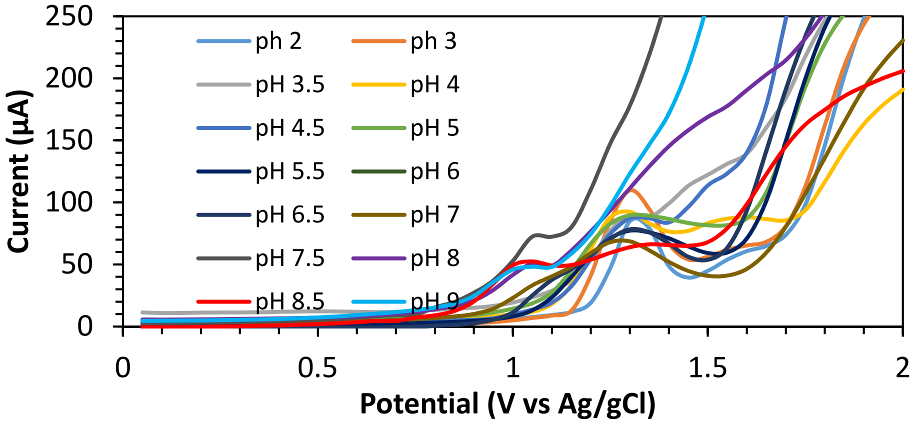

(b)

Figure S3. SWV curve of various pH (PBS 0,1 M range pH from 2-9) using **(a)** BDD and **(b)** BDD/NiNP.

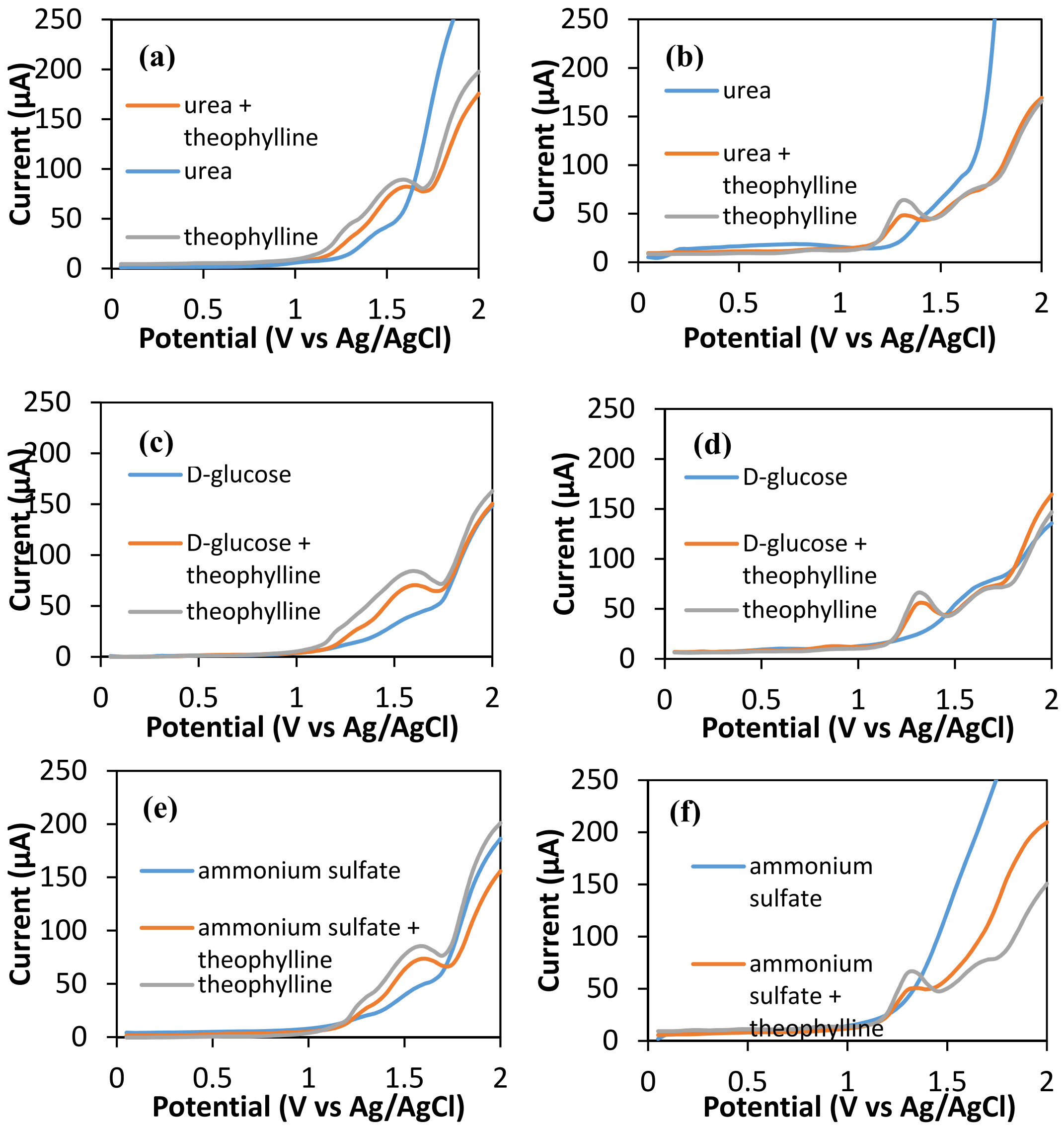

Figure S4. SWV curves of selectivity theophylline with urea interference using **(a)** BDD and **(b)** BDD/NiNP, D-glucose interference in **(c)** BDD and **(d)** BDD/NiNP, ammonium sulfate interference in **(e)** BDD and **(f)** BDD/NiNP.

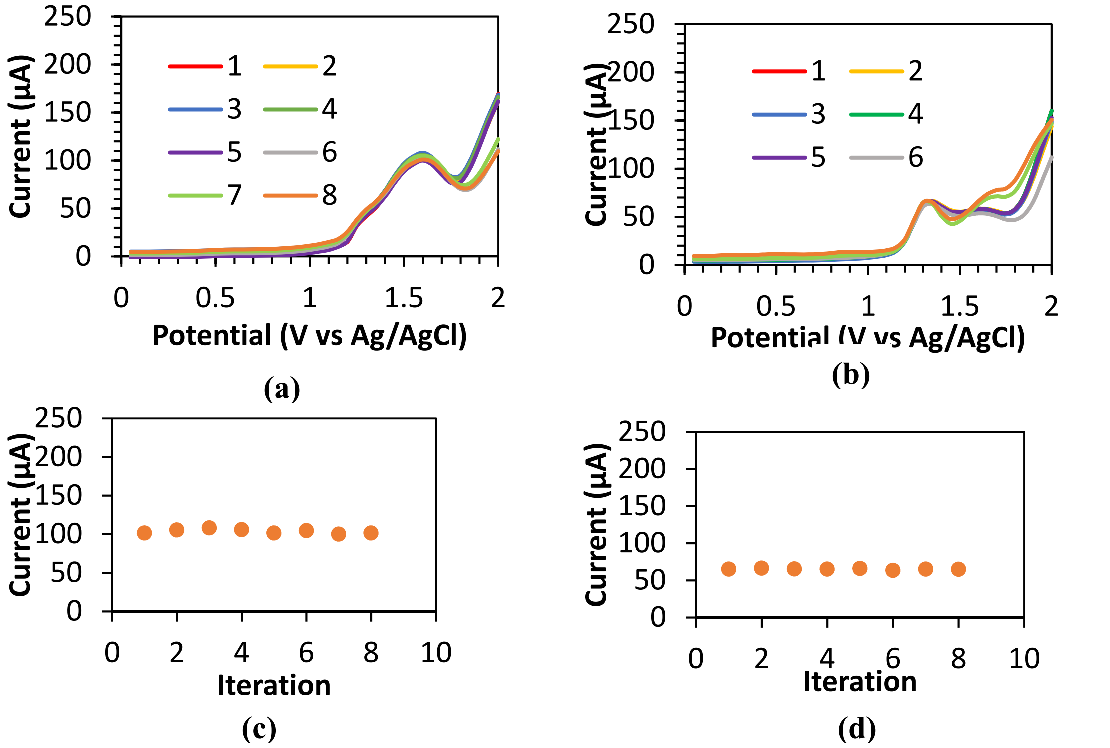

Figure S5. SWV curves for 60 µM theophylline with PBS 0.1 M pH 3 (n = 8) using **(a)** BDD and **(b)** BDD/NiNP. The plot of iteration against the peak current of theophylline using **(c)** BDD and **(d)** BDD/NiNP.
